# EHMTI-0386. Chronic subdural hematoma and spinal cerebrospinal fluid leak and in non-geriatric patients

**DOI:** 10.1186/1129-2377-15-S1-J16

**Published:** 2014-09-18

**Authors:** J Beck, CT Ulrich, J Gralla, J Fichtner, C Fung, W Z'Graggen, A Raabe

**Affiliations:** 1 Neurosurgery, Inselspital, Bern, Switzerland; 2 Neuroradiology, Inselspital, Bern, Switzerland

## Introduction

The etiology of chronic subdural hematoma (cSDH) in non-geriatric patients (≤60 years) often remains unclear.

## Aim

The primary objective of this study was to identify spinal cerebrospinal fluid (CSF) leaks in non-geriatric patients with the hypothesis that spinal CSF leaks are causally related to cSDH.

## Methods

All consecutive patients ≤ 60 years who were operated upon for cSDH from Sept. 2009 to April 2011 were included in this prospective cohort study. The patient workup included an extended search for a spinal CSF leak using a systematic algorithm: magnetic resonance (MR) imaging of the spinal axis with or without intrathecal contrast application, myelography/fluoroscopy, and postmyelography CT scanning. Spinal pathologies were classified according to direct proof of CSF outflow from intra- to extrathecal space, presence of extrathecal fluid accumulation, presence of spinal meningeal cysts, or no pathological findings.

## Results

Twenty-seven patients (mean age±SD: 49.6±9.2 years) were operated upon for cSDH. The chief complaint was headache in 15 (56%) patients. Hematomas were unilateral in 20 and bilateral in seven. In seven of 27 patients (25.9%) spinal CSF leakage was proven, in nine (33.3%) patients spinal meningeal cysts in the cervico-thoracic region were found, and three (11.1%) had spinal cysts in the sacral region. The remaining eight (29.6%) patients showed no pathological findings.

## Conclusions

Spinal imaging results are challenging the pathogenetic concept of cSDH in young patients. The direct proof of spinal CSF leakage in 25.9% of patients suggests that spinal CSF leaks may be a frequent cause of non-geriatric cSDH.

No conflict of interest.

